# Mortality in first- and second-generation immigrants to Sweden diagnosed with type 2 diabetes: a 10 year nationwide cohort study

**DOI:** 10.1007/s00125-020-05279-1

**Published:** 2020-09-26

**Authors:** Louise Bennet, Ruzan Udumyan, Carl Johan Östgren, Olov Rolandsson, Stefan P. O. Jansson, Per Wändell

**Affiliations:** 1 Department of Clinical Sciences in Malmö, Lund University, Malmö, Sweden; 2 Department of Family Medicine, Lund University, Malmö, Sweden; 3 Clinical Epidemiology and Biostatistics, School of Medical Sciences, Örebro University, Örebro, Sweden; 4 Department of Health, Medicine and Caring Sciences, General Practice, Linköping University, Linköping, Sweden; 5 Department of Public Health and Clinical Medicine, Family Medicine, Umeå University, Umeå, Sweden; 6 Institution of Medical Sciences, University Health Care Research Center, Örebro University, Örebro, Sweden; 7 Department of Public Health and Caring Sciences, Uppsala University, Uppsala, Sweden; 8 Department of Neurobiology, Care Sciences and Society, Karolinska Institutet, Huddinge, Sweden

**Keywords:** All-cause mortality, Cause-specific mortality, First-generation, Immigrants, Incident, Non-Western, Second-generation, Survival, Type 2 diabetes

## Abstract

**Aims/hypothesis:**

Non-Western immigrants to Europe are at high risk for type 2 diabetes. In this nationwide study including incident cases of type 2 diabetes, the aim was to compare all-cause mortality (ACM) and cause-specific mortality (CSM) rates in first- and second-generation immigrants with native Swedes.

**Methods:**

People living in Sweden diagnosed with new-onset pharmacologically treated type 2 diabetes between 2006 and 2012 were identified through the Swedish Prescribed Drug Register. They were followed until 31 December 2016 for ACM and until 31 December 2012 for CSM. Analyses were adjusted for age at diagnosis, sex, socioeconomic status, education, treatment and region. Associations were assessed using Cox regression analysis.

**Results:**

In total, 138,085 individuals were diagnosed with type 2 diabetes between 2006 and 2012 and fulfilled inclusion criteria. Of these, 102,163 (74.0%) were native Swedes, 28,819 (20.9%) were first-generation immigrants and 7103 (5.1%) were second-generation immigrants with either one or both parents born outside Sweden. First-generation immigrants had lower ACM rate (HR 0.80 [95% CI 0.76, 0.84]) compared with native Swedes. The mortality rates were particularly low in people born in non-Western regions (0.46 [0.42, 0.50]; the Middle East, 0.41 [0.36, 0.47]; Asia, 0.53 [0.43, 0.66]; Africa, 0.47 [0.38, 0.59]; and Latin America, 0.53 [0.42, 0.68]). ACM rates decreased with older age at migration and shorter stay in Sweden. Compared with native Swedes, first-generation immigrants with ≤ 24 years in Sweden (0.55 [0.51, 0.60]) displayed lower ACM rates than those spending >24 years in Sweden (0.92 [0.87, 0.97]). Second-generation immigrants did not have better survival rates than native Swedes but rather displayed higher ACM rates for people with both parents born abroad (1.28 [1.05, 1.56]).

**Conclusions/interpretation:**

In people with type 2 diabetes, the lower mortality rate in first-generation non-Western immigrants compared with native Swedes was reduced over time and was equalised in second-generation immigrants. These findings suggest that acculturation to Western culture may impact ACM and CSM in immigrants with type 2 diabetes but further investigation is needed.

**Figure:**
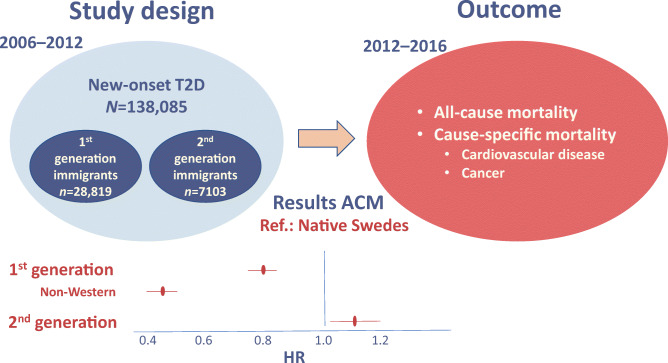
Graphical abstract

**Electronic supplementary material:**

The online version of this article (10.1007/s00125-020-05279-1) contains peer-reviewed but unedited supplementary material, which is available to authorised users.

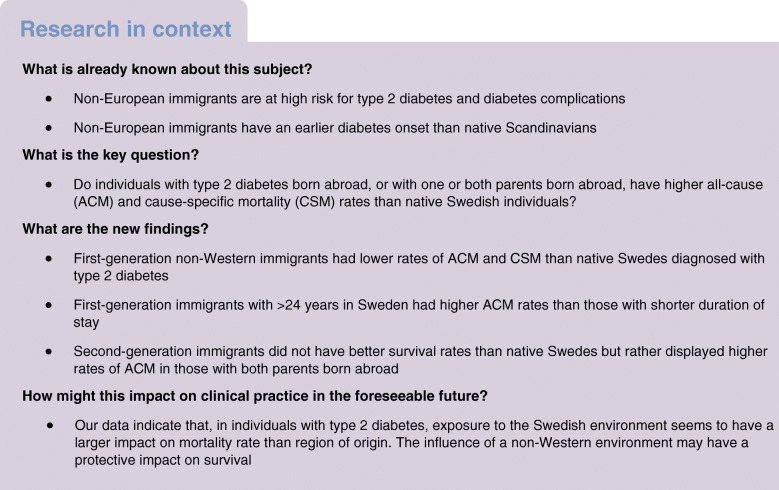



## Introduction

Type 2 diabetes is a complex chronic metabolic disease affecting a large proportion of the global population, particularly populations originating from South Asia, the Middle East and North Africa [1]. Type 2 diabetes influences the cardiovascular, renal and nervous systems, and contributes to increased morbidity due to macro- and microvascular complications and, subsequently, premature death [2].

Non-Western immigrants to Europe constitute a growing proportion of the recipient countries, and are at very high risk for developing type 2 diabetes [3, 4]. This increased risk is often attributed to genetic factors including epigenetics [5, 6], but environmental factors such as unfavourable lifestyle, obesity, poor socioeconomic situation, and cultural and social norms [7, 8] contribute even more [9]. A poor socioeconomic situation is strongly associated with poor lifestyle, e.g. intake of energy-dense foods and drinks, and a sedentary lifestyle [8]. A large proportion of non-Western immigrants to Sweden live in socioeconomically vulnerable neighbourhoods [1]. A previous Swedish longitudinal cohort study showed that refugees to Sweden referred to live in socioeconomically vulnerable areas developed diabetes to a greater extent than those referred to live in less vulnerable areas, reflecting the strong impact of socioeconomic status on type 2 diabetes risk [10].

Data based on the Swedish National Diabetes Register (NDR) have shown that although non-Western immigrants with type 2 diabetes attend more visits to their doctors, their metabolic control is worse and the risk of diabetic complications such as nephropathy is higher [11]. This indicates that mechanisms other than access to healthcare services influence disease trajectories. Interestingly, data from the NDR have paradoxically shown that non-Western first-generation immigrants had lower mortality rates than native Swedes [12].

Population-based studies of immigrant populations in Europe show a mortality advantage in first-generation immigrants that over time converges to the level of natives [13–15]; however, we have not found any studies of people with type 2 diabetes comparing mortality in first- and second-generation immigrants with natives.

In this study including all people with type 2 diabetes in Sweden identified through the Swedish Prescribed Drug Register, we aimed to compare all-cause mortality (ACM) and cause-specific mortality (CSM) rates between first-generation immigrants from different regions and native Swedes. Our secondary aim was to investigate the impact of time since migration on ACM. Our third aim was to compare ACM and CSM rates in second-generation immigrants and native Swedes.

## Methods

### Study population and data sources

Individuals with incident type 2 diabetes were identified from the Swedish Prescribed Drug Register, which was initiated in July 2005 [16]. The study population selection procedure has been described in a previous publication [17]. Briefly, people with type 2 diabetes were included if they initiated a glucose-lowering pharmacological treatment for type 2 diabetes dispensed at Swedish pharmacies sometime between 1 July 2006 and 30 June 2012. In correspondence with previous studies, people diagnosed between 30 and 75 years of age were included in the study [12, 18].

The Prescribed Drug Register was also used to identify and classify the received glucose-lowering treatments using Anatomical Therapeutic Chemical (ATC) classification codes by the WHO.

The LISA (Swedish acronym for Longitudinal Database of Education, Income and Occupation) register [19] was used to obtain information on Swedish vs non-Swedish background, whereby participants were classified into: (1) ‘native Swedes’, defined as those who, along with their parents, were born in Sweden; (2) ‘first-generation immigrants’ if born outside of Sweden to foreign-born parents; or (3) ‘second-generation immigrants’ if born in Sweden to one or two foreign-born parents. The LISA register also provided information on the country of birth, the highest educational level, disposable income, occupation and municipality of residence at diabetes diagnosis. The Swedish Cause of Death Register [20] provided information on the date and underlying cause of death, the latter recorded until 2012. Information on migration date and type of migration (immigration or emigration) was obtained from Statistics Sweden [1].

### Definition of non-Western origin

Individuals born in the Middle East, Asia, Africa, Latin America or the Caribbean were considered as having non-Western origin. Individuals born in the other countries but Sweden (reference) were considered as having Western origin.

### Definition of type 2 diabetes

Incident cases of diabetes were classified as ‘type 1’ or ‘type 2’ on the basis of glucose-lowering treatment received within 1 year after diagnosis date (electronic supplementary material [ESM] Table 1). Diabetes was classified as type 1 if treatment was by rapid-acting insulin (A10AB) solely or in combination with intermediate- or long-acting insulin (A10AC, A10AE).

Diabetes was classified as type 2 if glucose-lowering medications (GLMs) listed under the ATC A10B code were prescribed solely or in combination with insulin (A10AB, A10AC, A10AE, A10AD) (ESM Table 1). Participants who only received intermediate- or long-acting insulin were considered non-insulin-dependent and were classified as having type 2 diabetes. Correspondingly, those treated with mixed-insulin (A10AD) with or without combination with rapid-, intermediate- or long-acting insulin (A10AB, A10AC, A10AE) were also classified as having type 2 diabetes.

### Outcome assessment

The primary outcome was time to ACM. Participants’ follow-up started 1 year after the date of diabetes diagnosis, defined as the date of the first dispensed glucose-lowering drug, and continued until date of emigration, death or study end (31 December, 2016), whichever came first. In a secondary analysis, we assessed cancer-specific (ICD-10 codes C00-C97) and cardiovascular disease (ICD-10 codes I00-I99)-related mortality. Individuals were followed for CSM until 31 December, 2012, since the information on the underlying cause of death was available until the end of 2012.

### Statistical analysis

Patient characteristics were tabulated by country of birth. Cox proportional hazards regression models with time since diagnosis in years as the underlying time scale were fitted to assess survival differences between immigrants and native Swedes. The multivariable fractional polynomials method [21] assessed the functional form of continuous variables in the log-hazard function. Test and plots of Schoenfeld residuals evaluated the proportional hazards assumption, which was satisfied for migration status and country of birth of both first- and second-generation immigrants.

Separate analyses were conducted to estimate HRs and 95% CIs for the associations between migration status and mortality in the entire study population, as well as between country of birth and mortality for first- and second-generation immigrants. First-generation immigrants’ country of birth was categorised into the following groups: Africa; EU countries including the UK (EU28) except Nordic countries; Europe; Nordic other than Sweden; the Middle East; Asia; Latin America; and North America. Second-generation immigrants’ country of origin was categorised on the basis of their parents’ country of birth into the following groups: Nordic; European but not Nordic; North American; and non-Western, with each group reflecting an increasing gradient of non-Western origin. If parents came from different regions, we emphasised the parent with the highest degree of non-Western origin. Therefore, an individual with one parent from Sweden and one from the Baltic countries was classified as ‘European but not Nordic’, and an individual with one parent from Germany and one from Iraq was classified as ‘non-Western’.

Multivariable Cox regression models were adjusted for age at diabetes diagnosis, sex, attained education, disposable income, occupational socioeconomic group, region of residence and type of diabetes treatment. Age was modelled using restricted cubic splines with three knots. Knot locations were based on Harrell’s recommended percentiles [22]. Attained education at diabetes diagnosis was categorised by duration into compulsory (up to 9 years), secondary (10–12 years) and post-secondary (more than 12 years). Municipality of residence was categorised into living in small cities (<200,000 inhabitants) and larger cities (≥200,000 inhabitants). Individuals’ disposable income was divided into quintiles for descriptive statistics and modelled using restricted cubic splines with three knots for multivariable analyses. Occupational socioeconomic group was categorised into clerks, office holders and officials; other occupational workers; and non-employed. Participants over 67 years of age were classified as retired. Type 2 diabetes treatment was classified into insulin monotherapy; sulfonylureas/repaglinide monotherapy; metformin monotherapy; insulin and tablets; metformin and sulfonylureas/repaglinide; metformin and other tablets; and other monotherapies or drug combinations (ESM Table 2).

Multiplicative interaction terms were added to the adjusted model to test whether associations between regions of birth of first-generation immigrants and ACM differ by sex, age at diabetes diagnosis, disposable income and type 2 diabetes treatment. We also present the marginal plots of the effects of age at diagnosis and disposable income on ACM by region of birth (Figs. 3, 4).

Further analyses assessed the role of age at immigration and duration of residence in Sweden for the first-generation immigrants using native Swedes as the reference population. Age at immigration to Sweden was categorised into ≤21, >21 to ≤28, >28 to ≤38 and >38 years according to quartiles of the distribution. Duration of stay in Sweden was classified into ≤24 and >24 years (median duration of residence of first-generation immigrants = 24.2 years).

All calculations were performed using STATA software version 14SE (StataCorp, College Station, TX, USA).

### Ethical considerations

The Regional Research Ethics Board in Uppsala, Sweden approved the study.

## Results

The analysis is based on 138,085 people with type 2 diabetes who fulfilled inclusion criteria. Over a total observation period of about 833,095 person-years (median follow-up = 6.0 years, maximum follow-up = 9.5 years), 14,614 people died.

Table 1 shows characteristics of the study groups according to region of birth. The mean age at type 2 diabetes diagnosis in native Swedes was approximately 4 years greater than in first-generation immigrants and 7 years greater than in second-generation immigrants (Table 1). The earliest diabetes onsets were observed in immigrants originating from Africa, Asia and the Middle East. In first-generation immigrants, socioeconomic vulnerability differed considerably according to region of origin; approximately 50% of individuals originating from Africa, the Middle East and Asia had disposable income in the lowest quintile, whereas less than 15% of individuals originating from Sweden were within this income level. Further, immigrants were to a higher extent (>40%) non-employed, with the highest rates in Middle Eastern immigrants. Paradoxically, the education level was higher in individuals of non-Swedish origin. In second-generation immigrants disposable income, as well as education level, was on a par with that of native Swedes.

**Table 1:** Characteristics of native Swedes and first- and second-generation immigrants to Sweden with type 2 diabetes diagnosed between 2006 and 2012 (*N* = 138,085) identified through the Swedish Prescribed Drug Register

Characteristic	Native Swedes	First-generation immigrants by geographical region of birth *n* = 28,819								Second-generation immigrants *n* = 7103			
		Africa	EU28 except Nordic^a^	Europe^b^	Nordic countries^c^	Middle East^d^	Asia^e^	North America^f^	Latin America^g^	Nordic countries	European but not Nordic	North America^f^	Non-Western^h^
n	102,163	2277	4590	4353	6860	6883	2378	146	1332	1950	4657	320	176
	Col %	Col %	Col %	Col %	Col %	Col %	Col %	Col %	Col%	Col %	Col %	Col %	Col %
Age at diagnosis, mean (SD)^i^	60.6 (9.9)	49.4 (9.6)	60.0 (10.0)	56.4 (10.0)	61.2 (9.1)	51.7 (9.5)	49.7 (10.2)	53.6 (11.0)	54.1 (10.2)	56.0 (9.2)	51.1 (9.9)	61.8 (8.6)	43.6 (13.0)
Women	39.7	33.2	41.8	45.0	46.2	37.2	54.1	37.7	46.0	37.5	38.2	39.1	40.3
Quintiles of disposable income													
1	14.4	53.4	27.9	38.7	24.0	60.1	47.3	34.2	34.2	16.3	18.6	15.9	21.0
2	20.1	17.1	20.9	22.9	23.5	17.3	17.8	18.5	20.8	17.3	17.9	15.3	15.9
3	21.1	13.4	18.5	18.9	20.1	10.8	15.4	14.4	19.6	20.4	18.7	19.1	21.6
4	21.8	10.4	16.4	13.0	18.2	7.1	11.4	19.2	15.5	22.2	21.7	22.5	19.9
5	22.7	5.7	16.3	6.5	14.3	4.6	8.1	13.7	9.9	23.8	23.1	27.2	21.6
Attained education at diagnosis													
Compulsory	32.9	30.6	24.4	37.8	40.6	40.5	33.1	5.5	28.5	30.7	19.6	30.0	14.8
Secondary	47.5	39.8	47.3	44.5	44.6	31.4	34.7	28.8	43.2	51.1	55.9	45.6	51.1
Post-secondary	19.6	29.6	28.3	17.7	14.8	28.2	32.3	65.8	28.2	18.3	24.5	24.4	34.1
Occupation													
Clerks, office holders, officials	23.8	13.9	20.2	9.4	14.7	18.4	18.5	29.5	16.5	26.1	32.3	23.8	43.2
Other occupational workers	23.4	33.1	17.1	25.4	20.5	19.0	31.7	15.8	35.7	33.2	33.4	23.4	23.9
Non-employed	27.9	49.1	38.1	49.5	37.8	57.1	45.2	43.2	39.4	31.4	29.7	29.4	27.8
Retired	24.9	3.8	24.5	15.8	27.0	5.5	4.5	11.6	8.4	9.3	4.6	23.4	5.1
Area of residence													
Small cities (<200,000 inh.)	89.1	54.8	68.9	69.3	87.1	64.9	68.4	78.8	68.9	85.1	83.0	83.4	67.0
Larger cities (≥200,000 inh.)	10.9	45.2	31.1	30.7	12.9	35.1	31.6	21.2	31.1	14.9	17.0	16.6	33.0
Glucose-lowering treatment received within 1 year of diagnosis													
Insulin monotherapy^j^	3.9	5.7	3.7	2.7	4.0	2.4	3.2	5.5	4.2	3.6	3.8	4.4	6.8
Sulfonylureas/repaglinide^k^	4.1	7.6	5.6	5.4	4.2	4.9	5.9	6.2	4.3	3.2	2.2	4.1	3.4
Metformin monotherapy^l^	72.8	60.5	71.0	73.8	71.5	72.5	68.1	64.4	66.4	72.6	70.8	68.8	63.1
Insulin + any of the tablet(s)	9.5	14.1	7.6	7.6	9.8	7.6	10.8	9.6	13.6	10.7	12.1	9.4	13.6
Metformin^l^ + sulfonylureas/repaglinide^k^	6.2	9.5	7.5	6.6	7.0	8.6	8.5	6.8	7.6	6.7	6.4	6.9	7.4
Metformin^l^ + any other tablet(s)^m^	2.8	2.2	3.4	3.1	2.7	3.1	2.4	6.2	2.9	2.5	3.6	5.0	3.4
Other combinations or monotherapies	0.7	0.4	1.2	0.7	0.9	0.9	1.1	1.4	1.1	0.6	1.1	1.6	2.3

^a^EU28 except Nordic includes Belgium, Bulgaria, Czech Republic, Germany, Estonia, Ireland, Greece, Spain, France, Croatia, Slovenia, Italy, Cyprus, Latvia, Lithuania, Luxembourg, Hungary, Malta, Netherlands, Austria, Poland, Portugal, Romania, Slovakia, UK

^b^Europe does not include EU28 and Nordic countries. Includes former Soviet Union, other former Yugoslavia, other Western Europe, other Eastern Europe

^c^Nordic countries except Sweden. Includes Finland

^d^Middle East includes Iraq, Iran, Syria, Turkey, other Middle Eastern countries

^e^Asia includes Central Asia, East Asia, Southeast Asia, South Asia and the Indian Subcontinent, West Asia and the Caucasus

^f^North America, Australia, New Zealand and Oceania

^g^Latin America and the Caribbean

^h^Non-Western includes the Middle East, Africa, Asia and Latin America

^i^Mean age at diagnosis: first-generation immigrants 55.7 years and second-generation immigrants 52.7 years

^j^ATC: A10A insulin & analogues

^k^ATC: A10BB*, A10BX02

^l^ATC: A10BA02

^m^ATC: A10BD03, A10BD05, A10BD10, A10BD07, A10BD08

Col %, column percent; Inh, inhabitants

**Fig. 1:**
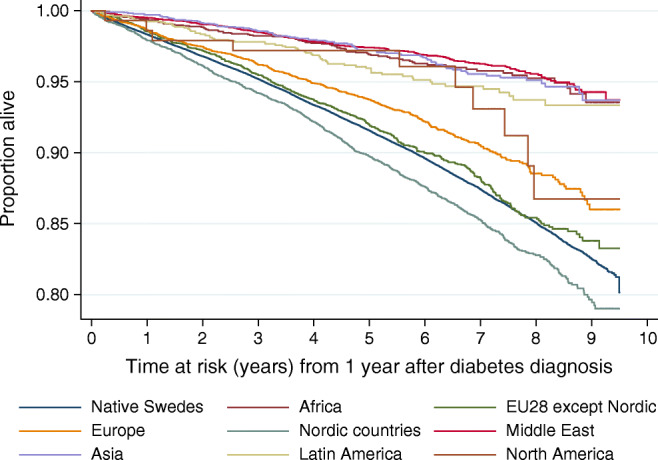
Kaplan–Meier survival estimates for native Swedes and first-generation immigrants with type 2 diabetes by their country/region of birth

Table 2 presents age-adjusted and multivariable-adjusted Cox regression analyses for ACM and CVD- and cancer-related mortality in relation to migration status, and country of birth of first-generation immigrants as well as parents’ country of birth of second-generation immigrants. Multivariable-adjusted analyses suggested lower ACM and CVD mortality rates for first-generation immigrants compared with native Swedes. Further, first-generation immigrants originating from Africa, Asia, the Middle East and Latin America had considerably higher overall survival rates (Fig. 1, Table 2) and lower CVD mortality rates (Table 2) than native Swedes. Immigrants born in the Middle East had considerably lower cancer-related mortality rates compared with native Swedes (Table 2). ACM and CSM rates in non-Western first-generation immigrants were considerably lower compared with native Swedes (HRs: ACM 0.46 [0.42, 0.50]; CVD 0.37 [0.27, 0.50]; and cancer-specific mortality 0.70 [0.55, 0.90]) (ESM Table 3). Mortality rates (ACM or CSM) did not differ between individuals of Western origin and native Swedes.

**Table 2:** HRs for all-cause, cardiovascular and cancer mortality in relation to migrant status and country/region of birth in individuals diagnosed with type 2 diabetes in Sweden between 2006 and 2012

Characteristic	ACM		Cardiovascular mortality		Cancer mortality	
	HR (95% CI)^a^	HR (95% CI)^b^	HR (95% CI)^a^	HR (95% CI)^b^	HR (95% CI)^a^	HR (95% CI)^b^
Entire study population						
Migrant status	*N* = 138,085		*n* = 125,052		*n* = 125,052	
Native Swedes	Reference	Reference	Reference	Reference	Reference	Reference
First-generation immigrants	0.93 (0.89, 0.97)**	0.80 (0.76, 0.84)***	0.95 (0.83, 1.09)	0.76 (0.66, 0.88)***	0.98 (0.87, 1.11)	0.96 (0.85, 1.09)
Second-generation immigrants	1.10 (1.01, 1.21)*	1.11 (1.02, 1.22)*	0.95 (0.70, 1.28)	0.97 (0.71, 1.31)	1.22 (0.96, 1.54)	1.23 (0.97, 1.56)
One foreign-born parent	1.07 (0.97, 1.18)	1.08 (0.98, 1.19)	0.99 (0.72, 1.37)	1.02 (0.74, 1.41)	1.17 (0.90, 1.51)	1.18 (0.91, 1.53)
Two foreign-born parents	1.28 (1.05, 1.56)*	1.28 (1.05, 1.56)*	0.69 (0.28, 1.66)	0.68 (0.28, 1.64)	1.52 (0.89, 2.58)	1.50 (0.88, 2.55)
First-generation immigrants by country of birth vs native Swedes						
Country of birth	*n* = 130,982		*n* = 118,667		*n* = 118,667	
Native Swedes	Reference	Reference	Reference	Reference	Reference	Reference
Africa	0.71 (0.57, 0.88)**	0.47 (0.38, 0.59)***	0.68 (0.34, 1.37)	0.37 (0.18, 0.76)**	1.21 (0.76, 1.93)	0.99 (0.62, 1.60)
EU28 except Nordic^c^	0.98 (0.90, 1.08)	0.90 (0.82, 0.99)*	1.05 (0.81, 1.37)	0.93 (0.71, 1.21)	0.97 (0.77, 1.24)	0.98 (0.77, 1.25)
Europe^d^	0.99 (0.89, 1.10)	0.83 (0.75, 0.92)***	0.83 (0.59, 1.18)	0.65 (0.46, 0.92)*	1.18 (0.91, 1.53)	1.17 (0.90, 1.53)
Nordic countries^e^	1.17 (1.09, 1.25)***	1.06 (1.00, 1.14)	1.26 (1.05, 1.53)*	1.12 (0.92, 1.36)	1.10 (0.91, 1.31)	1.07 (0.89, 1.28)
Middle East^f^	0.55 (0.48, 0.63)***	0.41 (0.36, 0.47)***	0.57 (0.38, 0.86)**	0.35 (0.23, 0.53)***	0.57 (0.40, 0.82)**	0.57 (0.40, 0.83)**
Asia^g^	0.66 (0.53, 0.81)***	0.53 (0.43, 0.66)***	0.43 (0.19, 0.97)*	0.32 (0.14, 0.72)**	0.75 (0.43, 1.30)	0.70 (0.40, 1.21)
Latin America^h^	0.66 (0.51, 0.84)***	0.53 (0.42, 0.68)***	0.57 (0.26, 1.28)	0.43 (0.19, 0.96)*	0.94 (0.55, 1.63)	0.82 (0.48, 1.43)
North America ^i^	0.89 (0.48, 1.65)	0.74 (0.40, 1.39)	N/A	N/A	0.65 (0.09, 4.62)	0.66 (0.09, 4.66)
Second-generation immigrants vs native Swedes						
Parents’ country of birth	*n* = 109,266		*n* = 99,121		*n* = 99,121	
Native Swedes	Reference	Reference	Reference	Reference	Reference	Reference
Nordic	1.09 (0.93, 1.27)	1.07 (0.91, 1.24)	0.66 (0.36, 1.24)	0.64 (0.34, 1.20)	1.50 (1.04, 2.16)*	1.48 (1.03, 2.13)*
European but not Nordic	1.07 (0.96, 1.21)	1.07 (0.95, 1.20)	1.00 (0.68, 1.46)	1.00 (0.68, 1.46)	1.15 (0.84, 1.58)	1.17 (0.85, 1.61)
North America^i^	1.16 (0.86, 1.56)	1.16 (0.87, 1.57)	1.45 (0.65, 3.23)	1.53 (0.69, 3.41)	0.74 (0.28, 1.98)	0.72 (0.27, 1.93)
Non-Western ^j^	1.03 (0.54, 1.98)	0.98 (0.51, 1.89)	N/A	N/A	N/A	N/A

^a^Adjusted for age at diagnosis

^b^Adjusted for age at diabetes diagnosis, sex, attained education, disposable income, occupational socioeconomic group, region of residence and type of diabetes treatment

^c^EU28 except Nordic includes Belgium, Bulgaria, Czech Republic, Germany, Estonia, Ireland, Greece, Spain, France, Croatia, Slovenia, Italy, Cyprus, Latvia, Lithuania, Luxembourg, Hungary, Malta, Netherlands, Austria, Poland, Portugal, Romania, Slovakia, UK

^d^Europe does not include EU28 and Nordic countries. Includes former Soviet Union, other former Yugoslavia, other Western Europe, other Eastern Europe

^e^Nordic countries except Sweden. Includes Finland

^f^Middle East includes Iraq, Iran, Syria, Turkey, other Middle Eastern countries

^g^Asia includes Central Asia, East Asia, Southeast Asia, South Asia and the Indian Subcontinent, West Asia and the Caucasus

^h^Latin America and the Caribbean

^i^North America, Australia, New Zealand and Oceania

^j^Non-Western includes the Middle East, Africa, Asia and Latin America

*p < 0.05, **p < 0.01, ***p < 0.001

N/A, not applicable

**Fig. 2:**
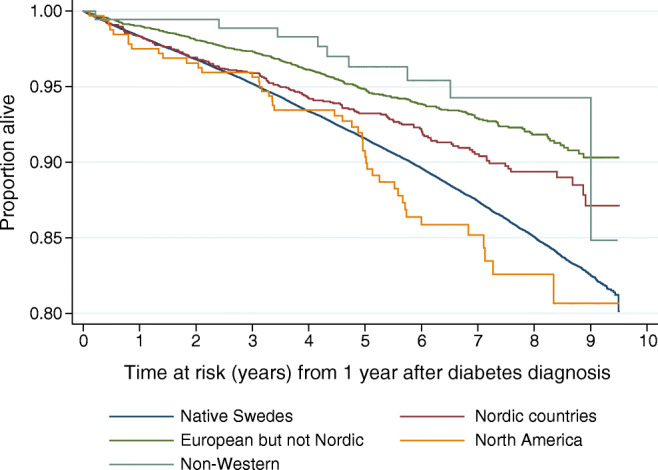
Kaplan–Meier survival estimates for native Swedes and second-generation immigrants with type 2 diabetes by their parents’ country/region of birth

While first-generation immigrants had lower all-cause and CVD mortality rates, second-generation immigrants had higher overall mortality rates than native Swedes (Table 2, entire population, Fig. 2). In the analyses classifying second-generation immigrants according to parents’ country of birth, the adjusted HRs were largely statistically non-significant, except for second-generation immigrants with parents born in the Nordic countries, presenting almost 50% higher hazards for cancer-related mortality (Table 2, parents’ country of birth-specific data).

Associations by age at immigration to Sweden of first-generation immigrants are displayed in Table 3, and by duration of residence in Sweden in Table 4. First-generation non-Western immigrants with older age at migration had generally higher-magnitude inverse association with ACM than those with younger age at migration (Table 3). Overall, higher magnitude inverse association was observed for first-generation immigrants with ≤24 years in Sweden than for those spending >24 years in Sweden (Table 4).

**Table 3:** Association between country/region of birth of first-generation immigrants and ACM among individuals diagnosed with type 2 diabetes in Sweden between 2006 and 2012 by age at immigration to Sweden

Variable	First-generation immigrants by geographical region of birth								All *n* = 28,819 HR (95% CI)
	Africa *n* = 2277 HR (95% CI)	EU28 except Nordic^a^ *n* = 4590 HR (95% CI)	Europe^b^ *n* = 4353 HR (95% CI)	Nordic countries^c^ *n* = 6860 HR (95% CI)	Middle East^d^ *n* = 6883 HR (95% CI)	Asia^e^ *n* = 2378 HR (95% CI)	Latin America^f^ *n* = 1332 HR (95% CI)	North America^g^ *n* = 146 HR (95% CI)	
Native Swedes	Reference	Reference	Reference	Reference	Reference	Reference	Reference	Reference	Reference
Age at migration (years)									
≤ 21	0.42 (0.16, 1.13)	1.05 (0.88, 1.25)	0.73 (0.50, 1.05)	1.11 (0.99, 1.23)	0.53 (0.33, 0.85)**	0.70 (0.33, 1.47)	0.32 (0.08, 1.29)	1.03 (0.26, 4.11)	1.02 (0.94, 1.11)
> 21 to ≤28	0.45 (0.29, 0.69)***	0.85 (0.72, 1.00)	0.78 (0.63, 0.97)*	0.99 (0.88, 1.12)	0.37 (0.26, 0.52)***	0.66 (0.43, 1.00)*	0.62 (0.33, 1.16)	0.23 (0.03, 1.63)	0.81 (0.75, 0.88)***
> 28 to ≤38	0.57 (0.42, 0.79)***	0.86 (0.71, 1.04)	0.96 (0.80, 1.15)	1.16 (1.00, 1.34)*	0.42 (0.33, 0.53)***	0.42 (0.28, 0.65)***	0.49 (0.32, 0.74)***	1.22 (0.39, 3.78)	0.79 (0.72, 0.86)***
> 38	0.39 (0.26, 0.58)***	0.86 (0.71, 1.03)	0.79 (0.67, 0.93)**	0.98 (0.81, 1.19)	0.40 (0.33, 0.48)***	0.53 (0.38, 0.73)***	0.57 (0.40, 0.80)**	0.85 (0.32, 2.27)	0.66 (0.60, 0.72)***

NOTE: Adjusted for age at diabetes diagnosis, sex, attained education, disposable income, occupational socioeconomic group, region of residence and type of diabetes treatment

^a^EU28 except Nordic includes Belgium, Bulgaria, Czech Republic, Germany, Estonia, Ireland, Greece, Spain, France, Croatia, Slovenia, Italy, Cyprus, Latvia, Lithuania, Luxembourg, Hungary, Malta, Netherlands, Austria, Poland, Portugal, Romania, Slovakia, UK

^b^Europe does not include EU28 and Nordic countries. Includes former Soviet Union, other former Yugoslavia, other Western Europe, other Eastern Europe

^c^Nordic countries except Sweden. Includes Finland

^d^Middle East includes Iraq, Iran, Syria, Turkey, other Middle Eastern countries

^e^Asia includes Central Asia, East Asia, Southeast Asia, South Asia and the Indian Subcontinent, West Asia and the Caucasus

^f^Latin America and the Caribbean

^g^North America, Australia, New Zealand and Oceania

*p < 0.05, **p < 0.01, ***p < 0.001

**Table 4:** Association between country/region of birth of first-generation immigrants and ACM among individuals diagnosed with type 2 diabetes in Sweden between 2006 and 2012 by duration of residence in Sweden

Variable	First-generation immigrants by geographical region of birth								All *n* = 28,819 HR (95% CI)
	Africa *n* = 2277 HR (95% CI)	EU28 except Nordic^a^ *n* = 4590 HR (95% CI)	Europe^b^ *n* = 4353 HR (95% CI)	Nordic countries^c^ *n* = 6860 HR (95% CI)	Middle East^d^ *n* = 6883 HR (95% CI)	Asia^e^ *n* = 2378 HR (95% CI)	Latin America^f^ *n* = 1332 HR (95% CI)	North America^g^ *n* = 146 HR (95% CI)	
Native Swedes	Reference	Reference	Reference	Reference	Reference	Reference	Reference	Reference	Reference
First-generation migrants									
≤ 24 years in Sweden	0.36 (0.27, 0.49)***	0.82 (0.68, 0.99)*	0.73 (0.62, 0.85)***	0.91 (0.73, 1.12)	0.37 (0.31, 0.43)***	0.40 (0.29, 0.55)***	0.51 (0.36, 0.71)***	0.65 (0.24, 1.73)	0.55 (0.51, 0.60)***
> 24 years in Sweden	0.62 (0.46, 0.83)**	0.91 (0.82, 1.01)	0.91 (0.79, 1.05)	1.08 (1.00, 1.16)*	0.48 (0.39, 0.60)***	0.68 (0.52, 0.91)**	0.55 (0.39, 0.78)***	0.81 (0.36, 1.79)	0.92 (0.87, 0.97)**

NOTE: Adjusted for age at diabetes diagnosis, sex, attained education, disposable income, occupational socioeconomic group, region of residence and type of diabetes treatment

^a^EU28 except Nordic includes Belgium, Bulgaria, Czech Republic, Germany, Estonia, Ireland, Greece, Spain, France, Croatia, Slovenia, Italy, Cyprus, Latvia, Lithuania, Luxembourg, Hungary, Malta, Netherlands, Austria, Poland, Portugal, Romania, Slovakia, UK

^b^Europe does not include EU28 and Nordic countries. Includes former Soviet Union, other former Yugoslavia, other Western Europe, other Eastern Europe

^c^Nordic countries except Sweden. Includes Finland

^d^Middle East includes Iraq, Iran, Syria, Turkey and other Middle Eastern countries

^e^Asia includes Central Asia, East Asia, Southeast Asia, South Asia and the Indian Subcontinent, West Asia and the Caucasus

^f^Latin America and the Caribbean

^g^North America, Australia, New Zealand and Oceania

*p < 0.05, **p < 0.01, ***p < 0.001

**Fig. 3:**
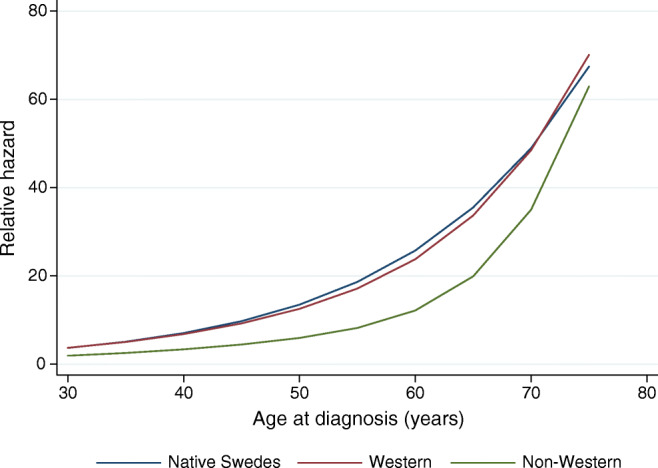
Marginal effect of age at diagnosis on the relative hazard of ACM in a Cox model by region of birth in the sample including native Swedes and first-generation immigrants diagnosed with type 2 diabetes in Sweden between 2006 and 2012. The model includes multiplicative interaction of region of birth and spline transformations of age, and multiplicative interaction of region of birth and spline transformations of disposable income, attained education, occupational socioeconomic group, region of residence and type of diabetes treatment

**Fig. 4:**
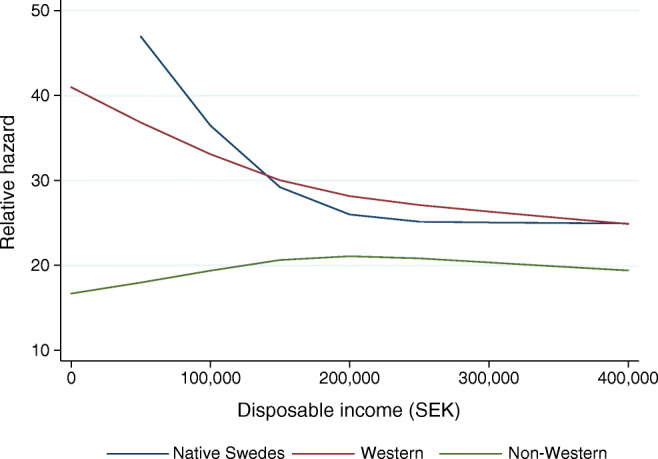
Marginal effect of disposable income on the relative hazard of ACM in a Cox model by region of birth in the sample including native Swedes and first-generation immigrants diagnosed with type 2 diabetes in Sweden between 2006 and 2012. The model includes multiplicative interaction of region of birth and spline transformations of disposable income, and multiplicative interaction of region of birth and spline transformations of age, attained education, occupational socioeconomic group, region of residence and type of diabetes treatment

The interaction terms with spline transformations of age and income were statistically significant (*p* values <0.001), suggesting that older age at diagnosis generally was associated with higher relative hazard, while higher disposable income was associated with lower relative hazard, although somewhat different shapes of functions were observed across region of birth of the first-generation immigrants (Figs. 3, 4).

The interactions terms with sex and type 2 diabetes treatment were not statistically significant (p values were 0.287 and 0.169, respectively), suggesting that the associations did not differ for men and women or across the treatment strategies.

## Discussion

To the best of our knowledge, this is one of the first studies investigating survival rates in first- as well as second-generation immigrants with type 2 diabetes. In this 10 year follow-up study, we show that first-generation non-Western immigrants have substantially lower ACM as well as CSM rates compared with native Swedes with type 2 diabetes. Further, we found that the younger the age at migration and the longer the duration of stay in Sweden, the larger the risk of shorter survival. For second-generation immigrants we did not observe beneficial survival rates compared with native Swedes, but rather, in particular in those with both parents born outside Sweden, they presented with shorter survival. Altogether, our study shows that first-generation non-Western immigrants at the early stage of migration initially are protected and display a mortality advantage over native Swedes, but, over time, in first-generation immigrants the lower mortality is subsequently reduced, and in second-generation immigrants is equalised.

The observed mortality advantage in first-generation immigrants has been reported previously. Data from the NDR report lower mortality rates in first-generation non-Western immigrants than the native Swedish-born population with diabetes [12]. Further, a Canadian long-term follow-up study, from 2005 to 2012, showed lower ACM and CVD-related mortality rates in first-generation immigrants with diabetes, an effect that persisted more than 10 years after immigration [23]. However, we have not found studies of mortality including first- as well as second-generation people with diabetes. Studies of the general population have shown that the health advantages in first-generation non-Western immigrants to Western countries erode over time [24, 25]. For instance, studies conducted in France, Belgium and Norway show lower ACM in first-generation immigrants, but, with duration of stay, the ACM converges towards that of natives [13–15], as we observe in our study. Contributing mechanisms may be connected to the influence of lifestyle, acculturation to the Western culture and epigenetics, but need further investigation.

The observed early diabetes onset and poor socioeconomic situation correspond with previous studies conducted in Sweden and Norway [26, 27]. It was previously shown that younger age at diabetes onset increases the risk of ACM [28], and that poor socioeconomic status with unemployment and lack of integration contributes to type 2 diabetes risk in non-Western immigrants to Sweden [9, 10]. Further, poor health literacy aggregates in socioeconomically vulnerable minority groups and is, according to the WHO, one of the most important determinants of health which contributes to high morbidity rates [29]. However, our data show important interactions among region of birth, age at onset and socioeconomic status (Figs. 3, 4). Our findings indicate that, in first-generation immigrants of non-Western origin, earlier age at onset, as well as low income, does not impact survival to as high an extent as in people with diabetes of Swedish or Western origin, and may partly explain the observed mortality paradox.

ACM in type 2 diabetes is mainly driven by complications in CVD [30]. Population-based studies have shown that Asian and Middle Eastern populations are not exposed to hypertension to the same extent as the native Nordic populations [31, 32]. The lower risk of hypertension may theoretically contribute to the observed lower CVD morbidity and mortality rates in the first-generation non-Western diabetes population, but this needs to be investigated further. Obesity, sedentary lifestyle and family history of diabetes are strong contributors to diabetes risk [33] and are highly prevalent in Middle Eastern and South Asian immigrant populations [3, 26]. It is reported that people diagnosed with mild obesity-related diabetes have lower risk of developing CVD-related complications [34]. Further, lower mortality rates are reported in obese people with type 2 diabetes, not only compared with the extremely obese, but also, paradoxically, compared with normal weight individuals with type 2 diabetes [35], indicating that ‘healthy obesity’ may influence the differences in mortality rates [36]. We have previously shown that first-generation Middle Eastern immigrants to Sweden present a more favourable fat profile than native Swedes, hypothetically protecting them from hypertension and CVD [37]. Thus, acculturation over time to Western lifestyle habits, with less-favourable fat intake, may contribute to the loss of mortality advantage in second-generation immigrants.

The beneficial cancer survival rates observed in first-generation non-Western immigrants (in particular, Middle Eastern immigrants) is consistent with previous register data on non-Western immigrants to Norway [38]. Insulin-resistant diabetes is reported to be associated with morbidity in cancer [39]. Hence, an explanation for the lower rates in non-Western immigrants may be related to type of diabetes, with lower prevalence of insulin-resistant diabetes in people of non-Western origin [3], but this remains to be further investigated.

One may argue that the ‘healthy migrant effect’, indicating that people migrating represent those most able to move and thus a healthier group compared with those not migrating [40, 41], may influence our findings. Although immigrants may have better health in relation to the conditions in their home country, their general mental and physical health in relation to the native Swedish population is generally not better [42]. We do not have data on the number of immigrants included in the study that have moved back and not returned; however, a database register study of refugees to Denmark from 1993 to 2010 did not show indications of remigration bias, but rather supported the opposite [41]. Due to the vulnerable political and economic situations that a large part of non-Western populations are exposed to, in combination with access to the Swedish health system where one gets treatment regardless of income or socioeconomic situation, we do not consider it likely that ‘salmon bias’ has influenced our data.

The strengths of this study are the sample size, the study design, the thorough sampling and the recent collection of data. The data were collected from national databases, including registers of drug prescription, socioeconomic status and mortality, and including all individuals in Sweden. Since socioeconomic vulnerability represents a strong determinant for migrant mortality from diabetes [43], all data in this study were adjusted for several variables reflecting socioeconomic burden. Although our data were adjusted for age at onset, we may not have been able to fully adjust for the large age difference (approximately 1.5 decades) between non-Western immigrants and native Swedes. Also, the older Swedish cohort may have a heavier cluster of comorbidities influencing survival rates. Our data lack information on metabolic control, comorbidities and related drug treatment, and lifestyle factors such as physical activity, diet, tobacco smoking and alcohol consumption impacting CVD risk and survival. We do not think this has influenced the outcome of our data since our results are consistent with previous data in Sweden on first-generation immigrants with type 2 diabetes [12]. Further, if the younger age at onset could explain differences in survival rates, we would expect similar results in second-generation immigrants as in first-generation immigrants, which we do not see. The power of a survival analysis is related to the number of events, and simulation work has suggested at least ten outcome events per predictor in the model [44], although sometimes this rule can be relaxed [45]. Due to the low number of events (ESM Fig. 4) in some subgroups, analysis of mortality is limited by low statistical power and so evidence is inconclusive in these groups.

The findings of this nationwide study of all people with type 2 diabetes in Sweden raise concerns regarding the erosion of life years to which non-Western immigrants with diabetes acculturating to the Western culture are exposed. From a clinical perspective, it is important to focus awareness on second-generation immigrants with diabetes to optimise non-pharmacological and pharmacological prevention to improve metabolic control and reduce the risk of diabetic complications. Future intervention studies of first- and second-generation non-Western immigrants are needed to increase the understanding of contributing mechanisms to mortality advantage, lifestyle and genetic contributions, and epigenetic interactions.

## Electronic supplementary material

ESM (PDF 84 kb)

## Data Availability

The data that support the findings of this study are available from the National Board of Health and Welfare, Statistics Sweden and the Swedish National Diabetes Register, but restrictions apply to the availability of these data, which were used under license for the current study and so are not publicly available.

## Abbreviations

ACM: All-cause mortality
ATC: Anatomical Therapeutic Chemical
CSM: Cause-specific mortality
EU28: European Union (EU) countries including the UK
LISA: Swedish acronym for Longitudinal Database of Education, Income and Occupation
NDR: National Diabetes Register
