# Supplementary material for: Mortality in first- and second-generation immigrants to Sweden diagnosed with type 2 diabetes: a 10 year nationwide cohort study
Source: Diabetologia. 2020 Sep 26;64(1):95–108. doi: 10.1007/s00125-020-05279-1 (PMC7716891; doi:10.1007/s00125-020-05279-1)
Supplement: Supplementary file 1 — (PDF 84 kb) [file 125_2020_5279_MOESM1_ESM.pdf]

ESM Table 1. Classification of type 1 and type 2 diabetes according to antidiabetic treatment received within 1 year after diagnosis date (date of first dispensed medication).

	A10B*<br>Glucose lowering medication	A10AB<br>Rapid acting insulin	A10AC/A10AE<br>Intermediate- or long lasting insulin	A10AD<br>Mix-insulin
Type 1 diabetes	no	**yes**	no	no
	no	**yes**	**yes**	no
Type 2 diabetes	no	no	no	**yes**
	**yes**	no	no	no
	**yes**	no	no	**yes**
	no	no	**yes**	no
	no	no	**yes**	**yes**
	**yes**	no	**yes**	no
	**yes**	no	**yes**	**yes**
	no	**yes**	no	**yes**
	**yes**	**yes**	no	no
	**yes**	**yes**	no	**yes**
	no	**yes**	**yes**	no
	**yes**	**yes**	**yes**	no
	**yes**	**yes**	**yes**	**yes**

*A10B are Glucose lowering medications including Metformin monotherapy (A10BA02), Metformin in combination with glitazones (A10BD03; A10BD05) or with DPP-4 inhibitors (A10BD07; A10BD08, A10BD10), Sulphonylurea (A10BB); Alpha glucosidase inhibitors (A10BF01); Glitazones (A10BG02; A10BG03); DPP-4 inhibitors (A10BH01, A10BH02, A10BH03); GLP-1RA (A10BJ01, A10BJ02, A10BJ03, A10BJ05, A10BJ06); Other glucose lowering medications (A10BX02, A10BX03)

ESM Table 2. Definition of treatment groups

**Treatment within 1 year of diabetes diagnosis**	Insulin monotherapy	SU/repaglinid	Metformin monotherapy	Metformin combination tablets	Glitazon	DPP-4	Glucobay	Other
Insulin monotherapy[a]	**yes**	no	no	no	no	no	no	no
Sulfonylureas/repaglinid [b]	no	**yes**	no	no	no	no	no	no
Metformin monotherapy [c]	no	no	**yes**	no	no	no	no	no
Insulin + **any** of the tablet(s)	**yes**	yes	yes	yes	yes	yes	yes	yes
Metformin [d] + sulfonylureas/repaglinid	no	**yes**	**yes**	**yes**	no	no	no	no
Metformin [d] +any other tablet(s)	**no**	yes	**yes**	**yes**	yes	yes	yes	yes
Other combinations or monotherapies	**no**	yes	**no**	**no**	yes	yes	yes	yes

Note: other tablets include ATC codes: A10BG02, A10BG03, A10BH01, A10BH02, A10BH03, A10BF01, other A10BX*

[a] ATC: A10A insulin & analogues

[b] ATC: A10BB*, A10BX02

[c] ATC: A10BA02

[d] ATC: A10BA02, A10BD05, A10BD03, A10BD10, A10BD07, A10BD08

ESM Table 3. All-cause mortality, cardiovascular mortality and cancer mortality in Western and non-Western first-generation immigrants vs native Swedes.

	All-cause mortality		Cardiovascular mortality		Cancer mortality	
	HR (95% CI) [a]	HR (95% CI) [b]	HR (95% CI) [a]	HR (95% CI) [b]	HR (95% CI) [a]	HR (95% CI) [b]
**Region of birth**	N= 130,982		n=118,667		n= 118,667	
Native Swedes	Reference	Reference	Reference	Reference	Reference	Reference
Western	**1.07 [1.02,1.13]**	0.96 [0.91,1.01]	1.10 [0.95,1.28]	0.95 [0.82,1.11]	1.07 [0.94,1.23]	1.06 [0.93,1.21]
Non-Western	**0.61 [0.55,0.67]**	**0.46 [0.42,0.50]**	**0.56 [0.42,0.77]**	**0.37 [0.27,0.50]**	**0.75 [0.59,0.95]**	**0.70 [0.55,0.90]**

[a] Adjusted for age at diagnosis

[b] Adjusted for age at diabetes diagnosis, sex, attained education, disposable income, occupational socio-economic group, region of residence, and type of diabetes treatment.

ESM Table 4. Mean age at death and number of deaths across the groups.

	Native Swedes	First generation immigrants by geographical region of birth n= 28,819								Second generation immigrants n= 7,103			
		Africa	EU28 except Nordic [a]	Europe [b]	Nordic countries[c]	Middle East [d]	Asia [e]	Latin America[f]	North America[g]	Nordic countries	European but not Nordic	North America [g]	Non-Western [h]
n	102,163	2,277	4,590	4,353	6,860	6,883	2,378	1,332	146	1,950	4,657	320	176
Age at death, mean (SD)[i]	70.5 (7.8)	62.8 (10.4)	70.0 (7.8)	68.0 (8.7)	69.8 (8.1)	64.0 (10.5)	63.4 (11.5)	64.7 (8.5)	67.8 (9.2)	65.4 (8.5)	61.4 (10.0)	70.9 (7.2)	56.8 (15.4)
All-cause mortality, n	11,810	86	495	372	932	237	89	66	10	165	299	44	9
CVD mortality, n	1,312	8	59	33	115	24	6	6	0	10	28	6	0
Cancer mortality, n	1,643	18	69	60	125	31	13	13	1	30	40	4	0

[a] EU28 except Nordic includes Belgium, Bulgaria, Czech Republic, Germany, Estonia, Ireland, Greece, Spain, France, Croatia, Slovenia, Italy, Cyprus, Latvia, Lithuania, Luxembourg, Hungary, Malta, Netherlands, Austria, Poland, Portugal, Romania, Slovakia, United Kingdom

[b] Europe does not include EU28 and Nordic countries. Includes former Soviet Union, other former Yugoslavia, other Western Europe, other Eastern Europe

[c] Nordic countries except Sweden. Includes Finland.

[d] Middle East includes Iraq, Iran, Syria, Turkey, other Middle Eastern countries

[e] Asia includes Central Asia, East Asia, Southeast Asia, South Asia and the Indian Subcontinent, West Asia and the Caucasus

[f] Latin America and the Caribbean

[g] North America, Australia, New Zealand, and Oceania

[h] Non-Western includes Middle East, Africa, Asia and Latin America

[i] Mean age of death: first generation immigrants 68.3 years and second generation immigrants 63.4 years.
